# Reduction of Melatonin Level in Patients with Type II Diabetes and Periodontal Diseases

**DOI:** 10.5681/joddd.2014.029

**Published:** 2014-09-17

**Authors:** Hamidreza Abdolsamadi, Mohammad Taghi Goodarzi, Fatemeh Ahmadi Motemayel, Mina Jazaeri, Javad Feradmal, Mahdiyeh Zarabadi, Mostafa Hoseyni, Parviz Torkzaban

**Affiliations:** ^1^Professor, Department of Oral Medicine, Dental Research Center, Hamadan University of Medical Sciences, Hamadan, Iran; ^2^Professor, Department of Biochemistry and Nutrition, Research Center for Molecular Medicine, School of Medicine, Hamadan University of Medical Sciences, Hamadan, Iran; ^3^Assistant Professor, Department of Oral Medicine, Faculty of Dentistry, Hamadan University of Medical Sciences, Hamadan, Iran; ^4^Department of Biostatistics and Epidemiology, School of Public Health, Hamadan University of Medical Sciences, Hamadan, Iran; ^5^Postgraduate Student, Department of Oral Medicine, Hamadan University of Medical Sciences, Haman, Iran; ^6^Student of Biochemistry and Nutrition, School of Medicine, Hamadan University of Medical Sciences, Hamadan, Iran; ^7^Associate Professor, Department of Periodontology, Dental Research Center, Hamadan University of Medical Sciences, Hamadan, Iran

**Keywords:** Iran, melatonin, periodontal disease, saliva, type II diabetes

## Abstract

***Background and aims.*** Melatonin is a circulating hormone that is mainly released from the pineal gland. It possesses antioxidant, free-radical scavenging, and immune-enhancing properties. A growing number of studies reveal a complex role for melatonin in influencing various diseases, including diabetes and periodontal diseases. The aim of this study was to examine the possible links between salivary melatonin levels and type II diabetes and periodontal diseases.

***Materials and methods.*** A total of 30 type II diabetic patients, 30 patients with periodontal diseases, 30 type II diabetic patients with periodontal disease and 30 age- and BMI-matched controls were studied. The periodontal status was evaluated by the Community Periodontal Index (CPI). Salivary melatonin levels were determined by a commercial enzyme-linked immunosorbent assay (ELISA) kit.

***Results.*** The mean of salivary melatonin level was significantly lower in patients with either periodontitis or diabetes compared to healthy subjects (P < 0.05). Salivary melatonin concentration decreased in type II diabetic patients and periodontitis patients, and then decreased reaching the lowest levels in type II diabetic patients with periodontal disease.

***Conclusion.*** Based on the results of this study, it can probably be concluded that salivary level of melatonin has an important role in the pathogenesis of diabetes and periodontal diseases. It is also worth noting that this factor could probably be used as a pivotal biological marker in the diagnosis and possible treatment of these diseases, although further research is required to validate this hypothesis.

## Introduction

Melatonin (N-acetyl-5-methoxy-tryptamine) is the main pineal hormone synthesized from tryptophan in a circadian manner. Melatonin has been recently recognized as a potent free radical scavenger and an immunomodulatory molecule. Besides the direct scavenging of free radicals, melatonin influences the oxidative stress status in an indirect way by stabilizing the inner mitochondrial membrane, improving the electron transport chain located there. Also, melatonin stimulates a number of antioxidative enzymes, including superoxide dismutase, glutathione peroxidase, glutathione reductase, and catalase. On the other hand, melatonin inhibits the pro-oxidative enzyme nitric oxide synthase.^1,2^ Furthermore, it has been shown that melatonin stimulates the proliferation and synthesis of type I collagen and promotes bone formation.^3^

The oxidative stress status is involved in the pathogenesis of some disorders such as periodontal disease and diabetes.^4,5^ Periodontal disease is an oral inflammatory process affecting the alveolar bone, gums and periodontal ligament. An important consideration in periodontal disease is the generation of free radicals and imbalance between the pro-oxidant and antioxidant systems. This status may lead to further oxidative attack and to substantial deterioration of periodontal tissues.^4^

The relationship between periodontal diseases and melatonin level remains unknown. However, melatonin may have implications in periodontal diseases by diminishing oxidative stress, limiting tissue damage, stimulating the immune response and reducing the progressive loss of alveolar bone.^6^

Not only is the pathology of periodontal disease connected with over-production of free radicals but also free radicals are involved in the pathogenesis of other disorders such as diabetes. Free radicals and oxidative stress are notorious for contributing to cell and tissue dysfunction and damage in diabetes. Hyperglycemia is the backbone of the pathophysiology of diabetes, leading to the development of complications through many intertwined cellular pathways like oxidative stress.^5,7^ On the other hand, melatonin is functionally linked to glucose metabolism in addition to having functions as an antioxidant and anti-inflammatory agent.^2,8^ Evidence exists that processes leading to and regulating the synthesis of the pineal hormone melatonin and the β-cell hormone insulin are dependent on one another in a largely unknown fashion. Most studies conclude that an increased insulin level in type II diabetic patients exerts an inhibitory effect on the pineal gland and melatonin, and a functional antagonism between insulin and melatonin has to be assumed. Such data suggest that the pineal gland and its melatonin-synthesizing machinery are sensitive to changes in insulin levels. Some results have shown that higher glucose and insulin levels are associated with lower melatonin levels in type II diabetic patients. Together, the above-mentioned principal components may constitute a hypothetical feedback-connection, which links the insulin- and melatonin-producing organs.^9-11^ Despite a large number of studies, the role of melatonin on glucose metabolism is rather controversial. Further investigations should be performed on animals and humans to clarify the role of melatonin on these disorders.

As mentioned above, there are reports that a lack of melatonin has effects on diabetes and periodontal diseases. According to the free radical theory in diabetes and periodontal disease, the present study was conducted to examine the relationship between melatonin levels as an antioxidant and diabetes, as well as the periodontal disease. To the best of our knowledge it is the first time melatonin level is measured in diabetic patients who suffer from periodontal disease.

We hope the measurement of melatonin levels in saliva will enhance our understanding of its role in etiopathology and pathophysiology of diabetes and periodontal diseases. In addition, melatonin level might probably be utilized as a potential marker in the diagnosis and treatment of diabetes and periodontal disease.

## Materials and Methods

### Subjects

This cross-sectional study was carried out at School of Dentistry, Hamadan University of Medical Sciences (Iran). A total of 120 subjects of both sexes (46 men, 74 women), aged 36 to 56 years (mean ± standard deviation = 45.7±8.5 years) were included in the study. The participants were fully informed about the study and gave written informed consent. The study protocol was approved by the University Ethics Committee and performed in accordance with the Code of Ethics of the World Medical Association according to the Declaration of Helsinki.

Dental and medical history of all the participants was in accordance with the criteria of the WHO.^12^ The patients underwent a clinical assessment and the details of age, gender, weight and height were obtained, with the BMI being calculated as the body weight in kilograms divided by the height in meters squared.

The subjects were divided into four age- and BMI-matched groups:

Group 1 (control) comprised 30 healthy subjects (18 women and 12 men, 45.9±8.29 years); all the healthy subjects were in good general health with no history of systemic disease or clinical signs of type II diabetes and periodontal disease.

Group 2 included 30 patients with type II diabetes (19 women and 11 men, 45.77±8.02 years),

Group 3 included 30 patients with periodontal disease (18 women and 12 men, 45.07±8.77 years).

Group 4 comprised 30 patients with type II diabetes and periodontal disease (19 women and 11 men, 46.33±9.25 years).

The inclusion criteria for patients with type II diabetes were: (a) age between 35 and 65, and FBS more than 126 mg/dL; (b) glycosylated hemoglobin (HbA1c) between 7.6 and 8.0% during the last 6 months, values compatible with a tolerable control of diabetes.^13^

The inclusion criteria for patients with periodontal disease were: age 35–65 years and evidence of periodontal disease. The periodontal diseases were diagnosed using the Community Periodontal Index.^12^ The Community Periodontal Index, currently recommended by the World Health Organization, consists of dividing the oral cavity into six sextants, with tooth indexing in each. Tooth indexes are 17/16 for the first sextant, 11 for the second, 26/27 for the third, 36/37 for the fourth, 31 for the fifth and 47/46 for the sixth. The teeth were examined using a probe with two marks located at 8.5 and 11.5 mm. The Community Periodontal Index codes used for recording periodontal status were as follows: code 0, healthy periodontium; code 1, moderate bleeding; code 2, presence of supra- or sub-gingival dental calculus; code 3, periodontal pocket of 4-5 mm; and code 4, periodontal pocket of 6 mm or higher. Subjects with CPI more than 0 were considered as having periodontitis. The same dentist performed all the examinations. A concordant diagnostic analysis was performed on 12 randomly selected patients by a second examiner, yielding an interobserver agreement coefficient of 81% for CPI assessments.

Exclusion criteria included having any other concomitant systemic disorders (such as epilepsy and schizophrenia), having diseases affecting the immune system and also receiving any medication other than for diabetes, which might alter melatonin levels.

Data were assessed by a single-masked examiner. The intra-examiner reliability was calculated at 84%.

### Saliva Collection

The patients and controls came to the School of Dentistry of the Medical University of Hamadan at 09:00 AM after 12-h overnight fasting. After 20 minutes of rest, a sample of saliva was obtained from each individual. In order to stimulate saliva production, the participants chewed a piece of paraffin wax for 7 min. Saliva produced during the first 2 minutes was discarded. Then, saliva was collected during the following 5 minutes to avoid any possible contamination. The patients chewed the paraffin during the time of saliva collection. Samples of saliva were centrifuged at 3000 rpm, 4ºC for 15 minutes, and then the clear supernatant was frozen at -80ºC until assays were performed.

### Salivary Melatonin Assay

The melatonin levels were analyzed in duplicate using commercially available ELISA kits (Direct Saliva Melatonin ELISA; EK-DSM, Switzerland), and the mean values of the duplicates were used for analyzing the results. The kit sensitivity was 0.5 pg/mL. The intra- and inter-assay coefficients of variation were 12.6% and 22.9%, respectively.

### Determination of FBS and HbA1c

All the participants reported to the laboratory at 09:30 AM and were seated for 30 minutes before sampling. Blood samples (5-7 mL) were collected from the antecubital vein and centrifuged at 3000 rpm for 10 minutes, followed by separation of the plasma fraction, which was then frozen (-20°C) until assay. The levels of glucose in plasma and HbA1c in whole blood were measured in all the study groups by BT-3000 autoanalyzer.

### Statistical Analysis

Data were analyzed by SPSS 16.0. ANOVA and post hoc Tukey or Dunnet tests were used to compare means and standard deviations of patient ages, CPI, salivary melatonin, FBS and HbA1c. The statistical significance of associations among variables was determined by using the Spearman correlation coefficient. Statistical significance was set at P<0.05.

## Results

Table 1 shows comparisons between patients and healthy individuals. All the groups of participants (46 males and 74 females) were matched for age and body mass index (BMI) (Table 1). The BMI was calculated according to the standard formula.

**Table 1:** Comparison of the study variables between the controls and patients

	Group 1	Group 2	Group 3	Group 4
Variable	(mean ± SD)	(mean ± SD)	(mean ± SD)	(mean ± SD)
Age (years)	45.9±8.29	45.77±8.02	45.07±8.77	46.33±9.25
BMI (kg/m^2^)	25.96±2.12	26.23±2.05	26.65±3.01	26.98±2.67
FBS (mg/dL)	110±8.00	160.87±31^*^	115±9	165.30±34.2^*^
Glycated Hemoglobin (%) HbA1c	6.1±1.00	7.74±1.4^*^	6.3±1	7.92±1.2^*^
CPI index	25.43±4.09	42.53±9.20^*^	51.77±10.42^*^	46.33±10.95^*^


In all the subjects, glucose, HbA1c and melatonin levels and also CPI index were assayed.

The comparison of serum levels of glucose and HbA1c between the patients and healthy controls is illustrated in Table 1. Patients with diabetes (groups 2 and 4) had significantly higher mean levels of glucose (160.87±31 and 165.30±34.2 mg/dL, respectively) (P<0.05), and HbA1c (7.74±1.4 and 7.92±1.2%, respectively) (P<0.05) than periodontitis patients and healthy subjects.

As expected, the CPI scores were significantly higher in patients with periodontitis (groups 2 and 3) than other groups (groups 1 and 3).

**Figure 1.**
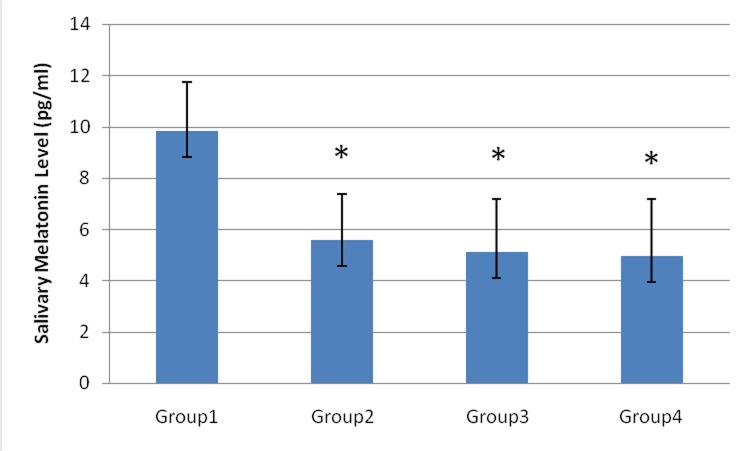
Salivary concentrations of melatonin (pg/mL) in group 1 (control): 30 healthy subjects (18 women and 12 men, 45.9±8.29 years); group 2: 30 type II diabetic patients (19 women and 11 men, 45.77±8.02 years); group 3: 30 patients with periodontal disease (18 women and 12 men, 45.07±8.77 years); and group 4: 30 type II diabetic patients with periodontal disease (19 women and 11 men, 46.33±9.25 years). (^*^) denotes significant differences compared to Group 1 (P<0.05).

The saliva melatonin concentrations were markedly lower in patient groups than in controls (P<0.05). In the current study, the mean saliva levels of melatonin were 9.8±1.9, 5.5±1.7, 5.1±2.1 and 4.9±2.2 pg/mL in the controls, type II diabetic patients, periodontitis patients and type II diabetic patients with periodontitis, respectively (Figure 1). Interestingly, the lowest level of salivary melatonin was observed in type II diabetic patients with periodontitis (group 4).

## Discussion

The subjects’ ages and BMI were matched; therefore, the effects of these variables on the results were eliminated. The rising of age and BMI correlated with the decline of melatonin,^14^ either due to normal age-dependent deterioration of the circadian pacemaker or neuronal transmission to the pineal gland, similar to that observed in neurodegenerative disorders^15^ or due to pineal gland calciﬁcation.^16^

In the present study, melatonin levels in saliva were determined; assessing salivary melatonin has recently been used as an alternative method for blood analysis since about 24–33% of the plasma melatonin appears in the saliva. Hence, the measurement of salivary melatonin levels represents an indirect, non-invasive procedure for the assessment of plasma melatonin levels which is very useful for the odontologist.^17^

Melatonin was selected as an assayed antioxidant in the current study since it has been shown that melatonin is more effective than classical antioxidants in reducing the oxidative stress.^18^

The aim of this study was to evaluate the salivary levels of melatonin as a likely marker in diabetes and periodontal disease. The study population consisted of a group of healthy subjects, a group of patients with type II diabetes, a group of patients with periodontal disease and a group of type II diabetic patients with periodontal disease. To the best of our knowledge it is the first time melatonin levels are determined in those with type II mellitus diabetes and periodontitis. The association between diabetes and periodontal diseases is well-established. Diabetes is a risk factor for periodontal disease, with diabetic patients exhibiting an increased prevalence, extent and severity of gingivitis and periodontitis compared to healthy adults.^19^

As expected, serum concentrations of glucose and HbA1c were significantly higher (P<0.05) in type II diabetic patients (groups 2 and 4) than in the controls and periodontitis patients (Table 1).

Data from this study indicated that the amount of serum melatonin secreted by salivary glands decreased in patients with diabetes and periodontitis. The present finding in keeping with the results of earlier research showed that melatonin may have anti-inflammatory effects probably due to its antioxidant and immune-enhancing action. Periodontal disease is well known to be associated with inflammation of the periodontium that destroys periodontal ligament and alveolar bone by resorptive processes. Increased oxidative stress in the inflamed area usually triggers the activity of the osteoclast and the bone resorption. Also, free radicals from the phagocytic cells migrating to the inflammation place damage the gingival tissues significantly.^6,20,21^

Previous data have shown an inverse relationship between per-oxidation products and the quantity of antioxidants in periodontal pathology. A key finding in periodontitis is polymorphonuclear neutrophil infiltration; these cells produce high amounts of free radicals. Moreover, a massive neutrophil migration to the gingiva and gingival fluid during periodontitis leads to abnormal spreading of free radicals.^4^

In this disease, an additional cause for the decline of melatonin may contribute or be even decisive. The toxic metabolite, 5-aminolevulinic acid, is a free radical-generating compound which leads to oxidative stress.^22^ It has been observed that high levels of oxidants can promote increases in the consumption of melatonin, even in organisms producing melatonin in concentrations by orders of magnitude higher than in vertebrates,^23^ and thus it may indicate that melatonin can be destroyed by free radicals generated at high rates.

The result of the current study showed low melatonin level in diabetic patients. To date very few studies about the inﬂuence of diabetic situations on the pineal gland exist and it is largely unclear why the melatonin plasma concentration is reduced under diabetic conditions. Noradrenaline is the main stimulus of pineal melatonin synthesis.^24^ Earlier investigations have illustrated that the pineal glands of diabetic animal model contain less noradrenaline and produce less melatonin in response to noradrenaline. The synthesis of melatonin starts with tryptophan, but the absolute amount of tryptophan is reduced in pineal glands of diabetic animals. Tryptophan deﬁciency can lead to decreased pineal and plasma melatonin concentrations. Other explanation for reducing melatonin in diabetes is that the expression of the melatonin synthesizing enzymes are altered under diabetic conditions and the concentrations of all the precursors are reduced in the pineal gland of diabetic animals.^25^

Similar to our results Cutando et al^26^found that the salivary melatonin level was lower in patients with diabetes and periodontitis. Although these two studies have the same results, there are differences between them such as matching the age and BMI of all participants which was performed in the present study. In the current study the FBS and HbA1c was measured and matched between two diabetic groups.

We assume that an increased insulin level in type II diabetes exerts an inhibitory effect on the pineal gland and melatonin, so a functional antagonism between insulin and melatonin has to be suggested.^27^ Complementary observations on type II diabetic patients displayed decreased melatonin plasma levels along with raised plasma insulin levels or increased pancreatic MT1- and MT2-receptor expression.^28^ The classic membrane associated melatonin receptors, in MT1 and MT2 mammals, have been found to be present in the pancreas and the islets of Langerhans. It has been determined that melatonin negatively affects insulin secretion through MT1- and MT2-receptors on the ß-cell surface.^11^,^29^ Together, the above-mentioned principal components might constitute a hypothetical feedback connection, which links the insulin- and melatonin-producing organs.

## Conclusion

To our knowledge, this is a first attempts to examine the relationship between salivary melatonin and its role in type II diabetes and periodontal diseases. Our findings demonstrated that melatonin level decreased significantly in patients with type II diabetes and periodontitis. Data from this study suggest the potential application of melatonin as a biomarker in the diagnosis and treatment of diabetes and periodontal disease. However, future studies with adequate sample power in this area are encouraged to validate the initial results reported here.

##  Acknowledgments

This study was performed based on a postgraduate thesis submitted to the Faculty of Dentistry, Hamadan University of Medical Sciences, in partial fulfillment of the requirements for the M.S. degree. Authors would like to thank the Vice Chancellor for Research and Technology of Hamadan University of Medical Sciences for supporting this study by a grant. We also would like to thank the Research Deputy of the Hamadan Medical School, whose extensive help at the Hamadan Dental School in the academic year of 2013 aided us in completion of this research. The authors declare no confilict of interests.
